# Facile fabrication of flexible metal grid transparent electrode using inkjet-printed dot array as sacrificial layer

**DOI:** 10.1038/s41598-022-05312-w

**Published:** 2022-01-28

**Authors:** Chaewon Kim, Kunsik An, Mingu Kang, Phillip Won, Jung-Jae Park, Kwan Hyun Cho, Seung Hwan Ko, Byeong-Kwon Ju, Kyung-Tae Kang

**Affiliations:** 1 Digital Transformation R&D Department, Korea Institute of Industrial Technology (KITECH), Sangnok-gu, Ansan-si, 15588 Korea; 2 Display and Nanosystem Laboratory, School of Electrical Engineering, Korea University, Seoul, 02841 Korea; 3 Department of Mechatronics Engineering, Konkuk University, Chungju, 27478 South Korea; 4 Applied Nano and Thermal Science Lab, Department of Mechanical Engineering, Seoul National University, 1 Gwanak-ro, Gwanak-gu, Seoul, 08826 South Korea

**Keywords:** Electronic devices, Surface patterning

## Abstract

In this study, we introduce a flexible metal grid transparent electrode fabricated using a lift-off process. This transparent electrode consisting of metal thin film with punched-like pattern by hole array was fabricated with 8 um separations. The separation of inkjet-printed etching resistant ink droplets was controlled in order to investigate the relationship between its electrical and optical properties of the electrodes. The aluminum areal density was defined to predict the electrical and optical properties of different arrays. A high and uniform transmittance spectrum appears to extend broadly into the UV region. The figure of merit of the transparent electrode was investigated in order to determine its performance as a transparent electrode. Moreover, there was no significant change in the resistance after 7000 bending cycles, indicating that the array conductor had superior stability. We also demonstrate transparent touch screen panels fabricated using the transparent electrode.

## Introduction

Transparent electrodes have been widely used in electronic devices such as solar cells^1^, displays^2^, touch screens^3^, thermoelectric devices^4^ and so on. Currently, transparent conducting oxides such as indium tin oxide (ITO) have been the most commonly used material because they offer high transmittance (> 80%) with low sheet resistance (≈ 15 Ω sq^-1^)^5^ and long reliability. However, indium as the main element of ITO is a rare earth material, resulting in high material cost^6^. Furthermore, metal oxides are also brittle and tend to crack and lead to device failure when deposited on flexible substrates. To overcome these limitations of transparent conducting oxides, many researchers have investigated alternatives for flexible transparent electrodes, such as sliver nanowires (Ag NWs)^7^, carbon nanotubes (CNTs)^8^, graphene^9^, conducting polymers^10^, metal oxide-metal–metal oxide structures (OMO)^11–13^ and metal grid patterns^14,15^ under the industrial demand for expansion of a form factor.

A metal grid pattern is a mesh structure of metal lines that enables not only high electrical conductivity but also high transparency through the area where the material is not deposited. This electrode type has advantages in stability compared to some other flexible conductors. For example, silver nanowires are one of the most promising candidates for alternative transparent electrode but it has severe disadvantages in chemical and thermal stability. Meanwhile, conducting polymers including PEDOT:PSS has exhibited high electrical conductivity with high transparency but they are weak in adhesion issues. E-beam lithography^16^, imprinting^17^, laser writing^18^, electrodeposition^19^ and inkjet printing have been utilized for patterning of the metal grid electrode. Among them, ink-jet printing, discharging of conductive ink (metal nanoparticles or a precursor solution) only on the area of interest, is a very promising technique because it has a low manufacturing cost due to a simple manufacturing process and small material consumption.^20–22^ The metal grid electrodes can be fabricated using various methods, such as lithography, soft lithography, printing.

The inkjet printing fabrication has many advantages but it also has a limitation in line width, which is determined by the minimum size of a printed drop. The drop size is concerned with ink viscosity, surface tension, printing conditions and so on, but generally 50 um to 100 um. This limitation restricts the usability of the electrode because it is visible to the naked eye.^22^ The wide width of the grid line results inconveniences users when the electrode within the device is facing the user, such as in a display application. To reduce its visibility to the naked eye, the line width should be less than 20 um, which is the minimum resolution of the human eye. Electrohydrodynamic (EHD) jet printing has also been studied to secure a narrow line width. This process provides a higher resolution than conventional inkjet printing by supplying voltage to the nozzle to form a strong electric field to print the solution. Jang et al.^23^ presented an invisible electrode by EHD jet printing with a line width less than 10 um. It was difficult to used at an industrial level in early stage because it is hard to maintain stable jetting condition under extremely high electric field between the nozzle and stages. In recent years, many researchers including Chen et al.^24^ and Khan et al*.*^25^ conducted an EHD study using multi-nozzle instead of single-nozzle. They developed the multi-nozzle printing system by restricting possible interference between electric fields by the voltage supplied to the nozzles. Likewise, the development of the fabrication process in large scale is being required in wide industrial area which is concerned with the flexible and transparent electrode.

In this study, we employed a transparent and flexible electrode using a negative pattern on an inkjet-printed dot array. The dot array pattern was fabricated using inkjet printing as a sacrificial layer. By removing the sacrificial layer, the metal layer deposited onto the sacrificial layer was removed together with the pattern of the punched hole array. This fabrication method is free from the line width limitation that enables invisibility to naked eye. Furthermore, using aluminum as the metal material instead of a noble metal significantly reduces the cost. The optical and electrical properties of the fabricated electrode were investigated by adjusting the drop distance and pattern array in the transparent electrode. Finally, the touch screen panel was made with the resistive type using the transparent electrode, which we successfully demonstrated.

## Methods

**Figure 1:**
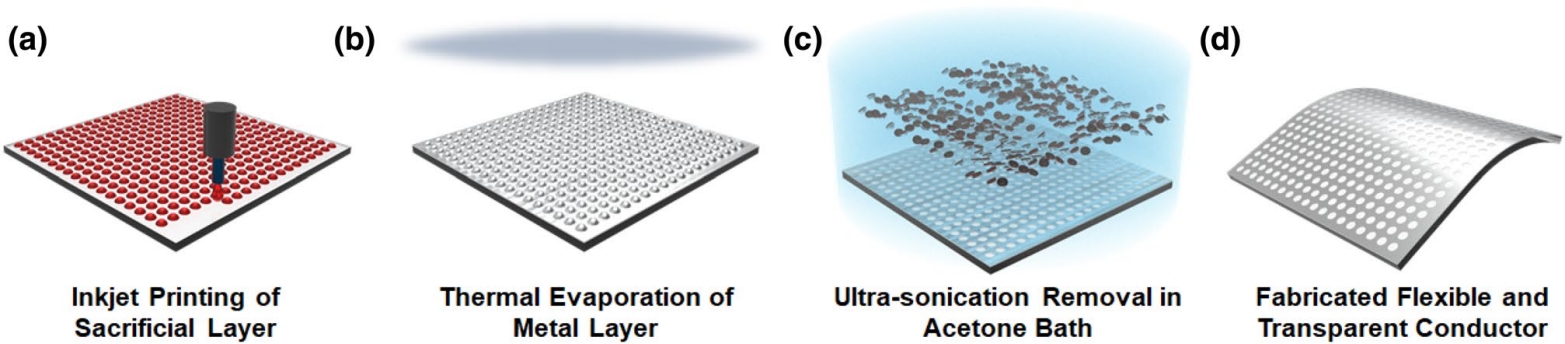
Schematic illustration for the fabrication procedure of a transparent electrode with a negative pattern of an inkjet-printed dot array. (**a**) PR ink-jet printing on the glass; (**b**) thermal evaporation of aluminum; (**c**) the removal of the printed PR array; (**d**) the metal grid electrode.

The polyethylene naphthalate (PEN) substrate was prepared for inkjet printing of the sacrificial layer. The substrate was cleaned thoroughly using ultrasonic sonication with acetone, isopropanol, and distilled water for 30 min respectively. After that, the ultraviolet surface treatment was performed for 20 min to improve adhesion between the ink and substrates. The detailed fabrication process of the transparent electrode is illustrated in Fig. 1. The ink was made by mixing a positive photoresist (PR) (az4330, AZ Electronic Materials) and its thinner (az1500, AZ Electronic Materials) with a 1:1 volume ratio using a voltex mixer. The blended solution has a viscosity of 9.15 cp at 24 °C and a surface tension 21.62 mN/m. A commercial inkjet printing system (Dimatix-2800, Dimatix) was used to fabricate a droplet array pattern. Each jetting of solution ink was maintained to a constant 10 pl using a cartridge head (DMP-11610, Dimatix) with a 19 um nozzle diameter. The nozzle voltage was adjusted within 28 ~ 31 V and the jetting frequency is 1 kHz. After being printed, the sample was baked at 110 °C for 80 s to increase adhesion between the substrate and sacrificial layer. The sample was exposed to extreme ultraviolet for 20 s. Then, 100 nm of aluminum was deposited on the sacrificial layer with thermal evaporation. Finally, the sacrificial layer was immersed in acetone and removed using ultrasonication for 20 min. Through the removal process of the sacrificial layer, the aluminum was patterned in the punched shape from the dot array.

## Results

**Figure 2:**
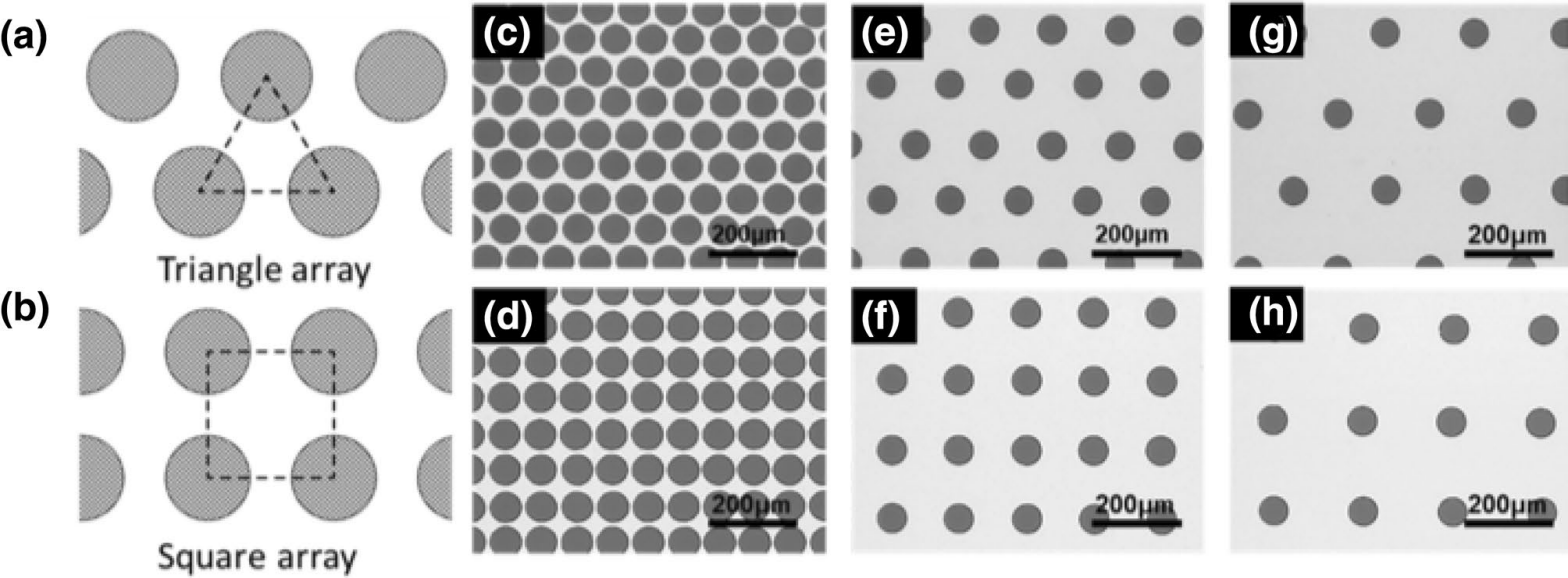
Pattern structure of the fabricated transparent electrodes with different patterning arrays and drop distances using a 67 um droplet. Schematic of the pattern shape that is connected by adjacent drop distances: (**a**) triangular shape, (**b**) square shape. Optical images by different droplet distances (**c**–**d**) 75 um, (**e**–**f**) 150 um, (**g**–**h**) 200 um.

The transparent electrode with a negative pattern of an inkjet-printed sacrificial dot array was fabricated with different array patterns and dot distances. The electrode was designed with two different arrays, a triangular array and a square array as explained in Fig. 2a,b. The drop spacing of the two array pattern varied from 75 to 200 um. The size of the printed droplets was consistently 67 um over the entire fabricated electrode. By decreasing the drop spacing, the width of the negative pattern could be decreased. For example, at 75 um drop spacing of the smallest distance, the width of the negative pattern was 8 um at the narrowest region. Since the negative region rather than the printed region was used, the width of the pattern could be decreased to make it narrower than the transparent part to the naked eye. Figure 2c–h present the optical microscope images of the fabricated transparent electrode in triangular and square shapes with 75 um, 100 um, and 150 um, respectively. The aluminum layer was colored light and the punched hole array was colored dark because the metal electrode reflected the incident light from the top. The hole pattern was formed precisely with the commercial inkjet system for printed electronics. The drop spacing could not be decreased more than 75 um because the printed drop was connected at a drop spacing of less than 75 um.

**Figure 3:**
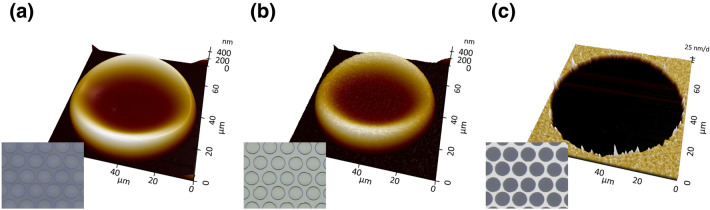
AFM images and microscope images (inset) of the single unit pattern at each step: (**a**) inkjet printing of the PR, (**b**) thermal evaporation of aluminum, and (**c**) removal of the sacrificial layer.

The fabrication steps of the transparent electrodes were studied through an optical microscope and atomic force microscopy (AFM). Figure 3 shows the steps of the fabrication process that corresponds to Fig. 1a,b,d. Figure 3a is an optical image of the patterned PR by ink-jet printing and an AFM image measuring the droplet shape of one of the patterns. There was a height difference in a printed droplet, which is known as the coffee ring effect because the shape is similar to a coffee stain mark. The coffee ring effect was formed from the difference in the relative evaporation rates of solvents during ink-jet printing.^26^ Since the evaporation rates of the printed ink at the outside was higher than that of the ink at the inside, the height of the edge was higher than that of the center. The ink used for the printing of the sacrificial layer also exhibited the conventional characteristics of the coffee ring effect. The maximum height of the edge area was 555 nm and the height of the center was 112 nm. After the sacrificial layer was printed, aluminum was deposited using thermal evaporation. One hundred nm of aluminum was deposited on the whole region of the printed sample, both for the sacrificial layer region and the bare substrate region. The study confirmed that the optical image was bright in the microscope image of Fig. 3b. The printed pattern followed the profile of the sacrificial layer with a concave center area. The aluminum electrode with a negative pattern dot array was fabricated by removing the underneath sacrificial layer. The patterned droplets were neatly removed by sonication in acetone, resulting in a transparent electrode with a negative pattern, as shown in Fig. 3c. The removal of the aluminum layer with the pattern of the underneath sacrificial layer was similar to the liftoff process in conventional lithography. In a general liftoff process, the thickness of the PR should be 3 ~ 4 times thicker than that of the metal layer because the height difference by the photoresist should be high enough to break the metal bonding. Though the thickness of the inkjet-printed sample was similar to the aluminum layer, the negative pattern was formed clearly and was attributed to the coffee-ring effect. Since the thickness of the edge was about 5 times higher than the center, the printed sacrificial layer gained the effect of a layer thick enough to break the metal bonding.

We measured the adhesion strength of the transparent electrode fabricated by tape test. Figure S1 shows that there was little change within 19 Ω/□ during peeling. Therefore, it confirmed that the adhesion between the substrate and the deposited aluminum was very good. Abrasive resistance test helps ensure that the material’s original structure and look are maintained. We measured the friction coefficient according to the distance by applying a load of 1 N and a speed of 1 cm/s through the Ball-on-Disc method. Through Figure S2, it was confirmed that the friction force of the fabricated transparent electrode was 0.947 N.

**Figure 4:**
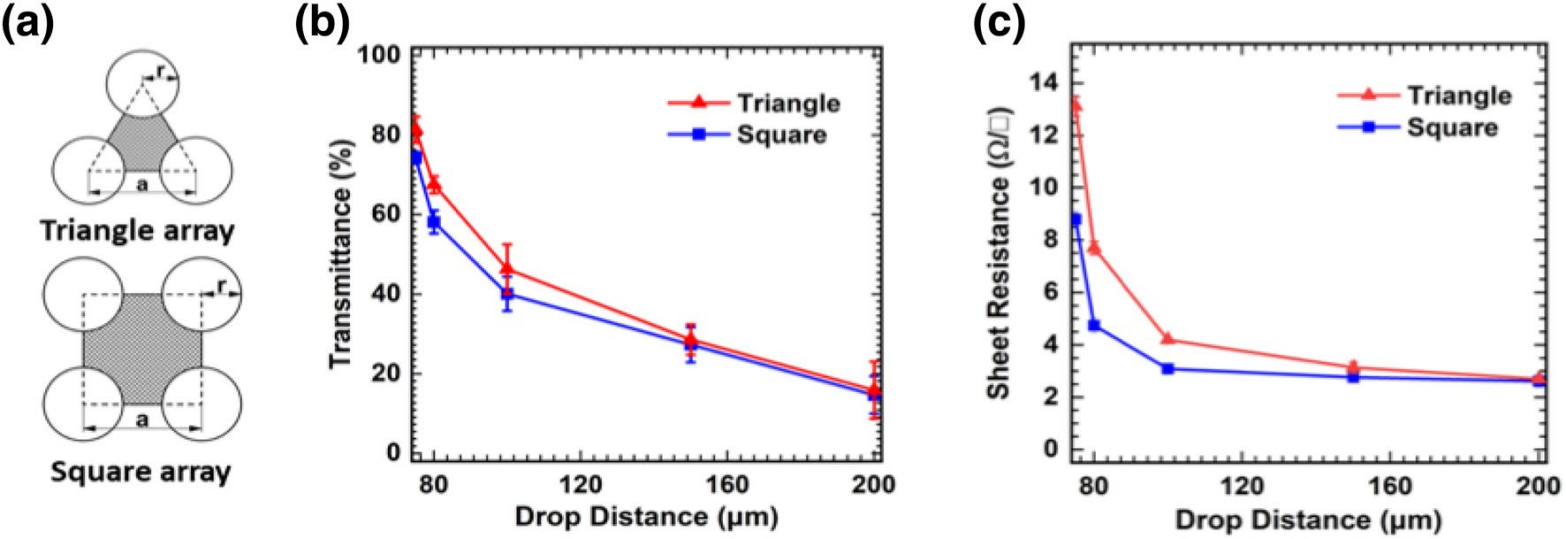
(**a**) Schematic illustration of the areal density of the aluminum area in the triangular and square array. (**b**) Transmittance at 550 nm of wavelength and (**c**) sheet resistance of the fabricated transparent electrode by drop distance with different drop pattern array.

Figure 4 shows the transmittance and the sheet resistance of the fabricated transparent electrode by the drop distance with the triangular and square array. The transmittance of the transparent electrode is gradually enhanced when the drop distance is decreased from 200 to 75 um, due to the decrease of the aluminum pattern area where the incident light was reflected. When the drop distance was 75 um, the maximum transmittance with a triangular array and square array achieved 81.4% and 74.4%, respectively. The optical properties are dependent upon the areal density of triangular and square arrays, which can be defined as the ratio of the aluminum area to the triangular and square areas. The areal density is expressed as:

1 $$\left( {\text{Triangle array}} \right){\text{Areal density}} = 1 - \frac{{2\pi r^{2} }}{{\sqrt 3 a^{2} }}$$

2 $$\left( {\text{Square array}} \right){\text{ Areal density }} = 1 - \frac{{\pi r^{2} }}{{a^{2} }}$$

where r is the drop radius, and a is the drop distance, as described in Fig. 4a. From the comparison of Eqs. (1) and (2), we estimated that the triangular array with a lower aluminum areal density had a higher transmittance than the square array. Comparing the calculated opening area with the measured transmittance, they exhibited similar tendency but they were not entirely consistent. It might attribute to a cause from the process to pattern the metal layer, there is inevitably over-etching or some error in the gap between the printed droplets.

Furthermore, the sheet resistance was also affected by the aluminum area density variation. As show in Fig. 4c, the sheet resistance gradually decreased, which increased the aluminum grid drop distance. Figure 4b,c indicate that the electrical conductivity and the transmittance had a trade-off relationship. An increase in the drop spacing created an increase in areal density, which increased the electrical conductivity and decreased the transmittance. From the same point of view, the sheet resistance of the square array was lower than that of the triangular array. Not only the drop distance, the characteristics of the electrode was also determined by the thickness. Figure S3 shows the transmittance and sheet resistance of the transparent electrode with various thicknesses. The aluminum pattern with the thickness higher than 100 nm was not achieved because the thickness is too thick to break the films along the topography of the printed PR. On the other hands, the transparent electrode with the thickness smaller than 100 nm exhibited small correlation with transmittance and large correlation with sheet resistance. Though the transmittance depends on the areal density of aluminum which was determined by not the thickness but electrode pattern, sheet resistance strongly depended on the thickness of the film. The decrease in the sheet resistance is more substantial compare with the decrease transmittance. The FoM as expressed in Eq. (3) is inversely proportional to the sheet resistance, confirming that the FoM improved as the thickness increased.

**Figure 5:**
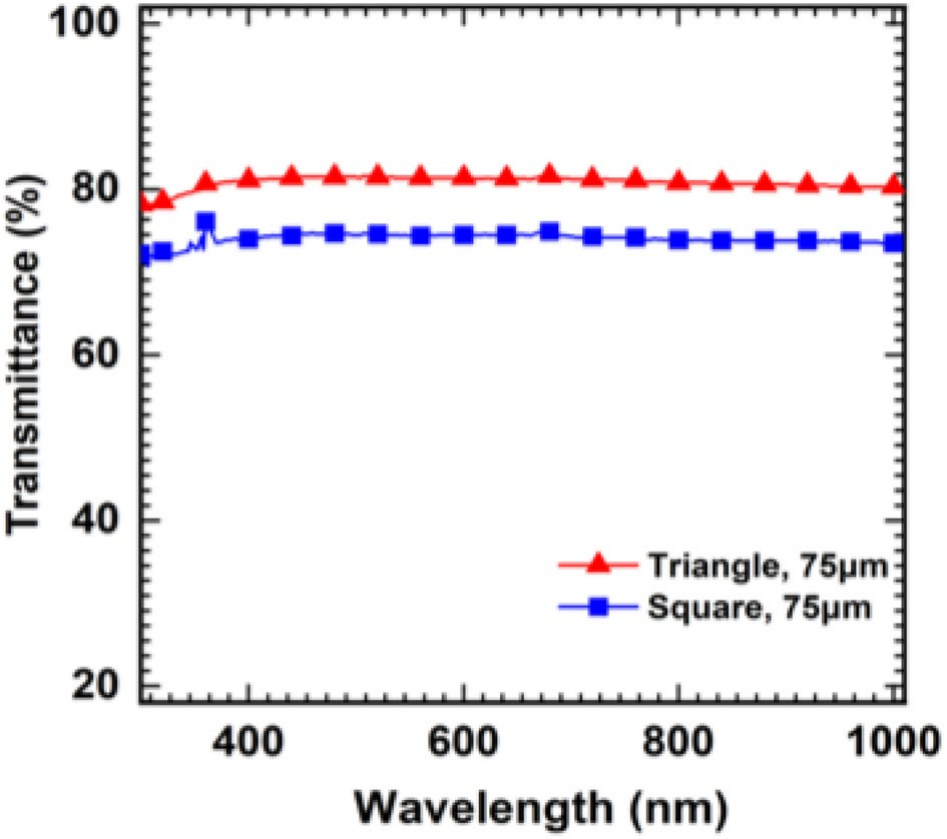
Total transmittance spectra of the fabricated transparent electrode with a 75 um drop distance.

The total transmittance (T) of the fabricated transparent electrode formed by the triangular and square array with 75 um drop spacing at wavelengths from 300 to 1000 nm is shown in Fig. 5. The samples with the other drop spacings were also measured as shown in Figure S1. The measurement area covered total 0.5 cm by 1 cm in area and there was negligible difference at repetitive measurements. The measurement was conducted with the setting based on the PEN film so transmittance of the PEN film was neglected. All of the samples exhibited the characteristics of uniform transmittance regardless of the wavelength, and the fabricated transparent electrode with the triangular and square array showed transmittances of T_400_ = 81.1%, T_900_ = 80.6%, S_400_ = 74.0% and S_900_ = 73.8%. The other measurement results with drop distances from 80 to 200 um are given in Figure S4, which also exhibits the uniform transmittance. Moreover, the transmittance in the visible range was slightly lower than that of conventional ITO, but the UV area transparent electrodes still had a high transmittance unlike ITO where light absorption occurred, which indicated that the fabricated transparent electrode had a broad transmittance spectrum. These characteristics are advantageous in optoelectronic devices because the electrodes do not deform the spectrum. The fabricated transparent electrode could also be used in optoelectronic devices in the ultraviolet (UV) and infrared (IR) regions as well as the visible region.

In general, transmittance and electrical conductivity has a trade-off relationship. To evaluate the fabricated electrode properly considering the relationship, the figure-of-merit (FoM) values were calculated. An evaluation index that included the transmittance and the sheet resistance, the FoM, was firstly suggested by Haacke^27^ as in Eq. (3) where T is the optical transmittance at a wavelength of 550 nm and $R_{S}$ is the sheet resistance (Ω $sq^{ - 1}$).

3 $${\text{FoM }} = \frac{{T^{10} }}{{R_{sheet} }}$$

**Figure 6:**
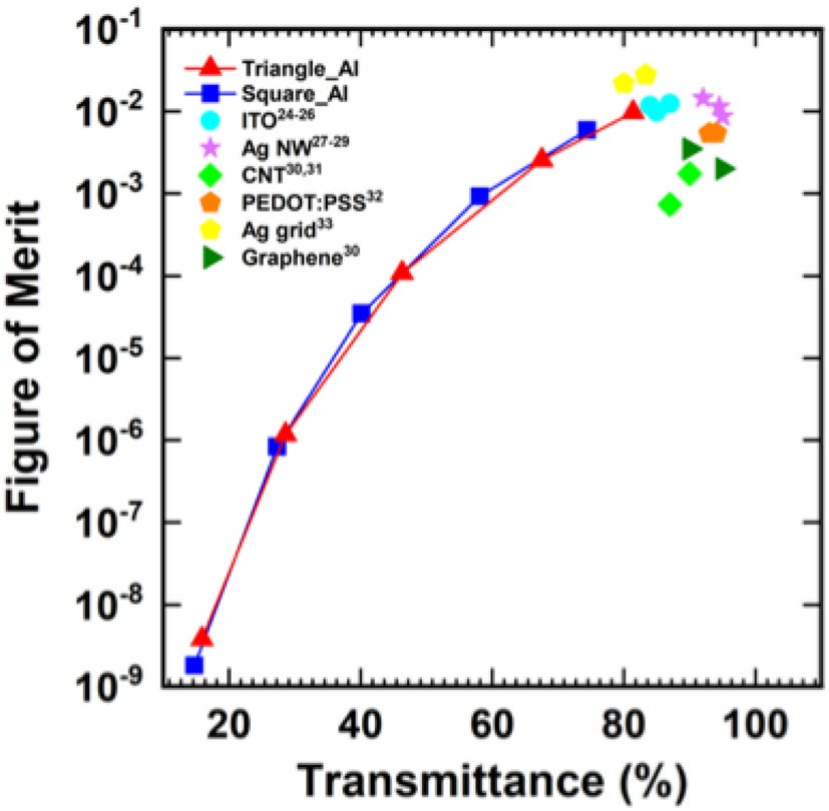
Figure-of-merit values of the fabricated transparent electrode compared with other types from the literature (ITO^25,26^, Ag NW^28–30^, CNT^31,32^, PEDOT:PSS^33^, Ag grid^34^, Graphene^31^) for comparison.

Figure 6 shows the relationship between the transmittance and the FoM of the fabricated transparent electrode. The FoM increased with an increasing transmittance; in other words, by decreasing the drop spacing. The maximum FoM was obtained at the minimum drop spacing of 75 um and the FoM of the electrode with a triangular array was 9.7 × 10^–3^ and that with a square array was 5.9 × 10^–3^. The FoM of the triangular array and that of the square array had the same relationship and the FoM was determined by the transmittance regardless of the array pattern. The calculated FoM was also compared with other materials for transparent electrodes. ITO^28–30^, AgNWs^31–33^, CNT^34,35^, PEDOT:PSS^36^, Ag grid^37^, Graphene^34^ were selected for the comparison that was suggested in both the literature and for commercial products. The fabricated transparent electrode exhibited comparable values of FoM with the fabricated electrode at over 80% of transmittance. Though the transparent electrode was fabricated using a very simple method, the performance was on a high level that brought the advantages of low fabrication cost. Furthermore, it required a small material cost and did not need expensive nanomaterials such as AgNWs, CNT, and graphene. The required amount of the photoresist for printing of the sacrificial layer was also small because the drop array was patterned with a drop-on-demand method with a high edge height.

**Figure 7:**
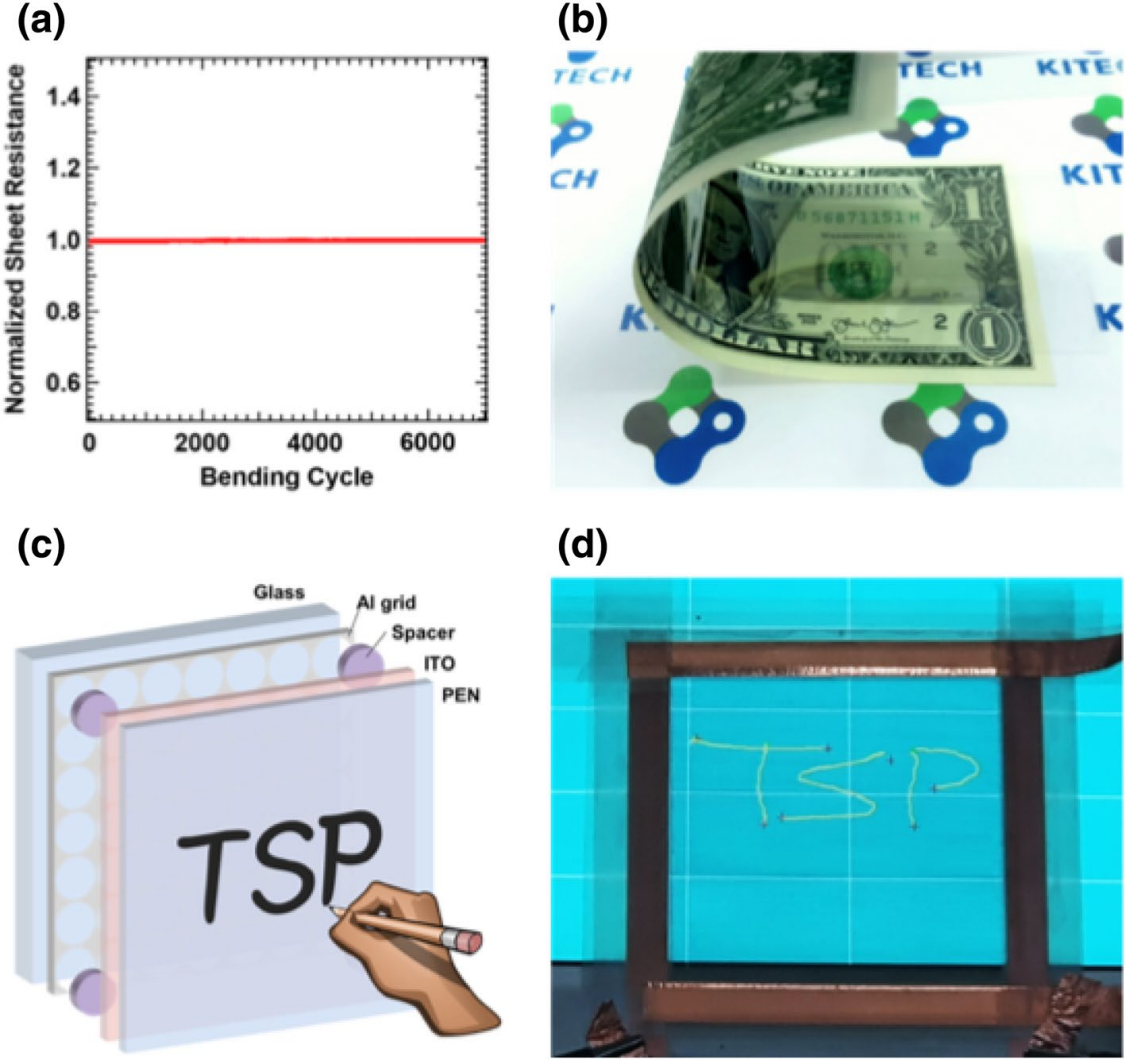
(**a**) Measured normalized sheet resistance during the mechanical bending test with a 5 mm bending radius. (**b**) Flexible transparent electrode demonstrated on a one-dollar bill. (**c**) Schematic illustration of a touch screen panel (TSP) (**d**) TSP operation on an LCD screen. “TSP” was written on TSP.

To determine the mechanical flexibility of the fabricated Al electrode, the electrode was folded back and forth with a bending radius of 9 mm to 0.5 mm, and its resistance was compared to its initial value. The resistance of the Al grid mesh electrode remained stable up to a bending radius of 1 mm, which indicates that the transparent electrode has excellent mechanical flexibility. The electromechanical reliability of the fabricated transparent electrode was plotted in Fig. 7a by measuring the normalized sheet resistance during the cyclic bending test. The test was performed with 1 Hz of repetition rate and 5 cm by 5 cm of sample size. The resistance was measured frequently with the rate of 4 times per cycle. There was little change within 1% during the 7000 cycles of the bending test. The electrode guaranteed the mechanical robustness against bending fatigue. In addition, we conducted bending tests on commercial ITO/PEN electrodes for further comparison. The resistance of the ITO/PEN electrode increased rapidly according to bending cycles, as shown Figure S5. These properties show a contrast in flexibility with our fabricated electrodes. Figure 7b shows the flexible electrode that was attached on the center of a one-dollar bill to exhibit its flexibility. The electrode has undergone cyclic bending test, and the normalized resistance of the electrode after each cycle in the relaxed state was plotted in Fig. 7a. The region of the transparent electrode could be seen at a slightly darker region of the bill rather than at the outside edges. A touch-screen panel (TSP) was demonstrated and is shown in Fig. 7c,d. The pattern of the triangular array with a 75 $\mu$ m drop spacing was selected for the demonstration. Figure 7c shows a schematic illustration for the demonstration of a four-wire resistive touch-screen panel. The TSP consists of the fabricated transparent electrode and the counter electrode of ITO deposited on a PEN substrate. While having the spacers in between these electrodes, the top conducting layer for resistive touch screen panel must be flexible because a mechanical pressure should be regularly applied, and the bottom electrode can be either rigid or flexible. ITO-PEN film is used as a counter electrode with comparable sheet resistance. The size of each drop pattern was 67 m$\mu$ for the substrate and the area of the fabricated transparent electrode was 10 cm $\times$ 8 cm. The resistive touch panel detects a position of a touch by capturing the electrical resistance at the point of contact where an electrical short is created. The copper tapes are applied at both ends of each conductor and properly insulated to measure accurate detection of touch. The function of the produced TSP was confirmed by writing the letters “TSP” on the screen. The letters were accurately recognized on the screen by detecting touch pressure on the fabricated TSP.

TSP was successfully demonstrated by using the fabricated transparent electrode with patterned aluminum thin film despite of the roughness of the electrode. The gap between the upper and lower electrode was far from the On the other hand, the non-uniform thin film might be disadvantages to the devices with the structure of stacked thin film such as thin film solar cells^38,39^ and light emitting diodes^40,41^. The roughness of the electrodes might result in electrical nonuniformity and leakage current sometimes in severe cases. The lack of flatness was remained as further issue to expand its usability. Meanwhile, this fabrication process had advantages in wide choice for the material. The experiments in this study choose the PR because it was easy to control the viscosity to be within the printable range. The selected solution was also clearly removed by the ultrasonication process with immersed in acetone. Therefore, if the solution satisfies these two conditions, any other commercial materials can be employed in this study. Not only the sacrificial material, metal for the thin film also be in wide choice such as copper as well as aluminum. The variability of the solution selection is one of great advantages on our studies.

## Discussion

A flexible transparent electrode was fabricated by patterning aluminum thin film though an inkjet-printed sacrificial layer. With this process, a metal grid electrode can be produced simply by inkjet-printing and thermal evaporation without a shadow mask. Furthermore, many kinds of material, including metallic and inorganic elements, can be used with this method, which expands its application. This facile fabrication method enabled fine patterning of the electrode with a width of less than 10 um, which is less than the visibility of the naked eye. Unlike the commercial ITO, it can be used in the UV region or IR region as well as the visible region. The figure-of merit, defined to optimize the transmittance and sheet resistance, was comparable to the other materials as candidates for flexible transparent electrodes. This transparent electrode also exhibited an excellent stability in any mechanical stress, including bending. Finally, a touch screen panel was demonstrated to confirm the potential of the transparent electrode on versatile applications. This work, including the new fabrication technology with a mask-less process for transparent electrodes, will provide new insights for the further development of thin film electronics.

## Supplementary Information

Supplementary Information.
