# Supplementary material for: Facile fabrication of flexible metal grid transparent electrode using inkjet-printed dot array as sacrificial layer
Source: Sci Rep. 2022 Jan 28;12:1572. doi: 10.1038/s41598-022-05312-w (PMC8799687; doi:10.1038/s41598-022-05312-w)
Supplement: Supplementary file 1 — Supplementary Information. [file 41598_2022_5312_MOESM1_ESM.docx]

**Supporting Information**

**Facile Fabrication of Flexible Metal Grid Transparent Electrode using Inkjet-printed Dot Array as Sacrificial Layer**

*Chaewon Kim^1,2,+^, Kunsik An^3+^, Mingu Kang^1^, Phillip Won^4^, Jung-Jae Park^4^, Kwan Hyun Cho^1^, Seung Hwan Ko^4^, Byeong-Kwon Ju^2*^ and Kyung-Tae Kang^1*^*

^1^ Digital Exchange Department, Korea Institute of Industrial Technology (KITECH), Sangnok-gu, Ansan-si 15588, Korea

^2^ Display and Nanosystem Laboratory, School of Electrical Engineering, Korea University, Seoul 02841, Korea

^3^ Department of Mechatronics Engineering, Konkuk University, Chungju 27478, South Korea

^4^ Applied Nano and Thermal Science Lab, Department of Mechanical Engineering, Seoul National University, 1 Gwanak-ro, Gwanak-gu, Seoul, 08826 South Korea

^*^ktkang@kitech.re.kr (Kyung-Tae Kang)

^*^bkju@korea.ac.kr (Byeong-Kwon Ju)

^*^these authors contributed equally to this work.

^+^these authors contributed equally to this work.


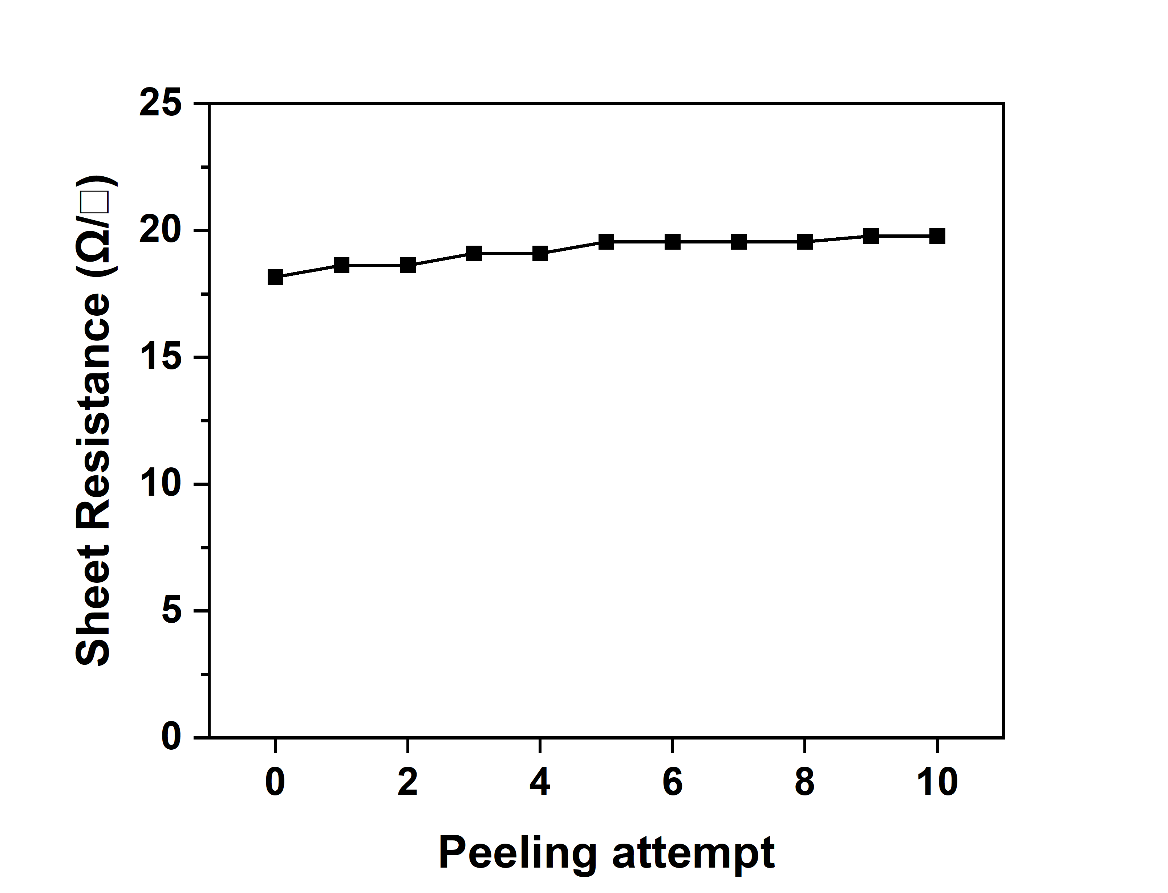


**Figure S1.** Measured sheet resistance by peeling attempt the tape for adhesive properties.


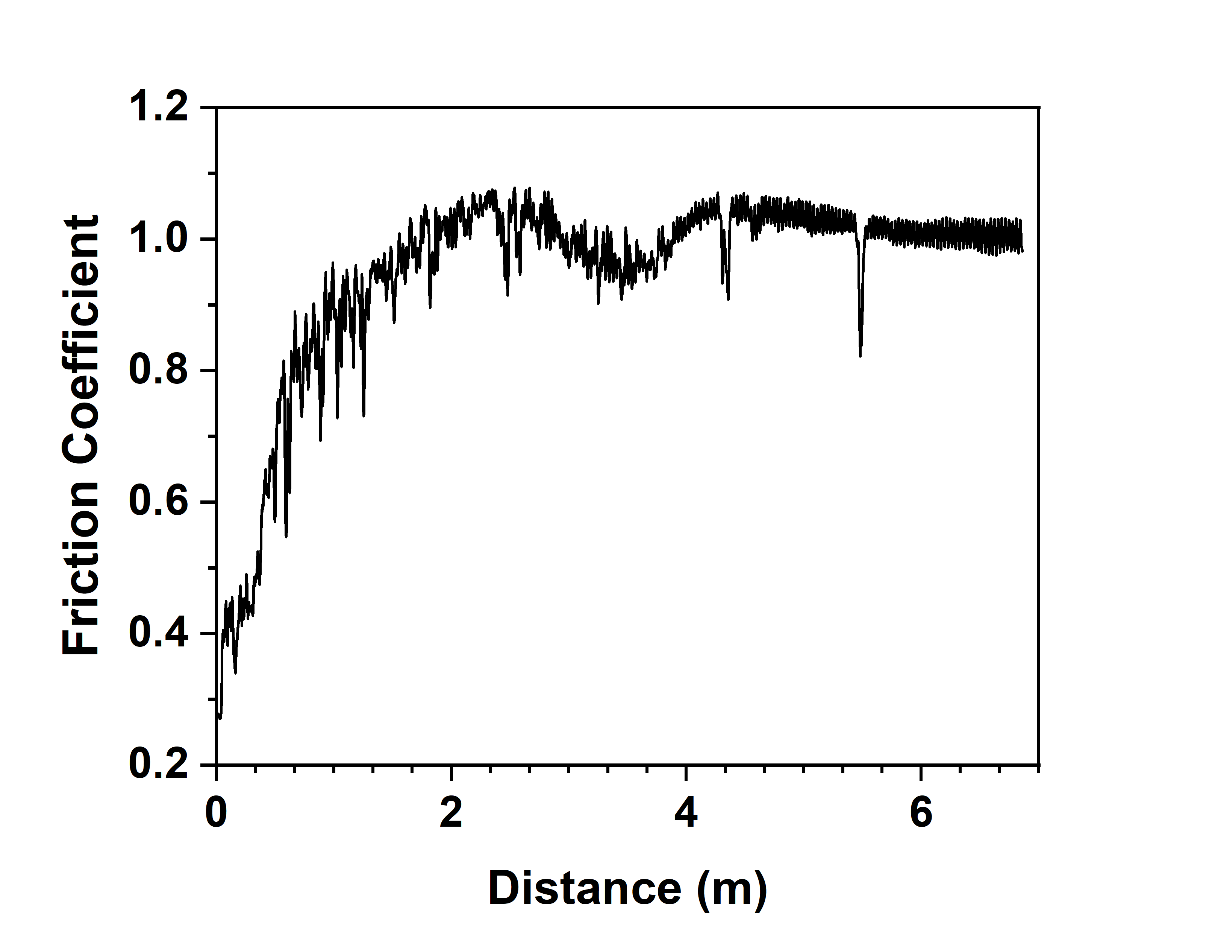


**Figure S2.** Measured the mean friction coefficient with distance by Ball-on-Disc.


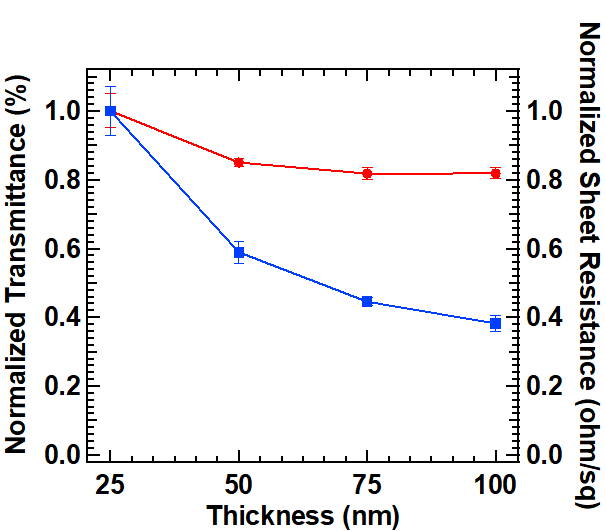


**Figure S3.** Transmittance and sheet resistance of the transparent electrode with various thickness from 25 nm to 100 nm.

**
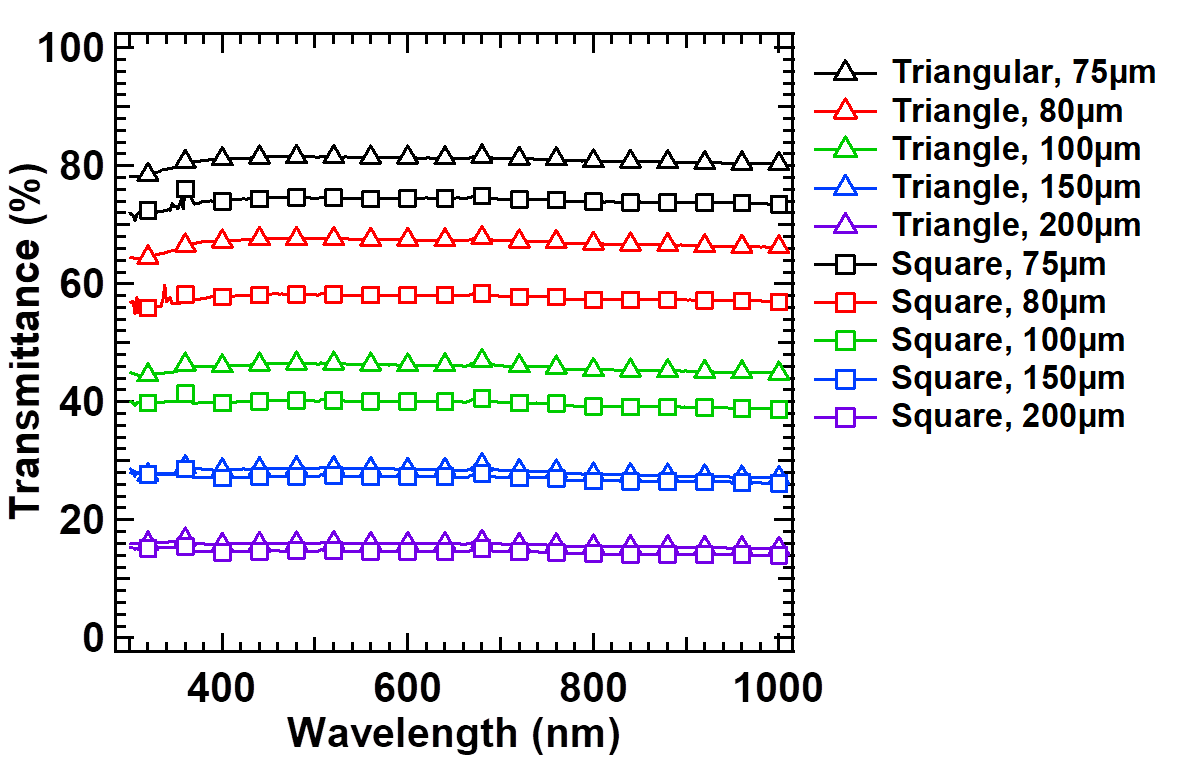
**

**Figure S4**. Total transmittance spectra of the fabricated transparent electrode in array pattern of triangle and square with drop distance from 75 um to 200 um.


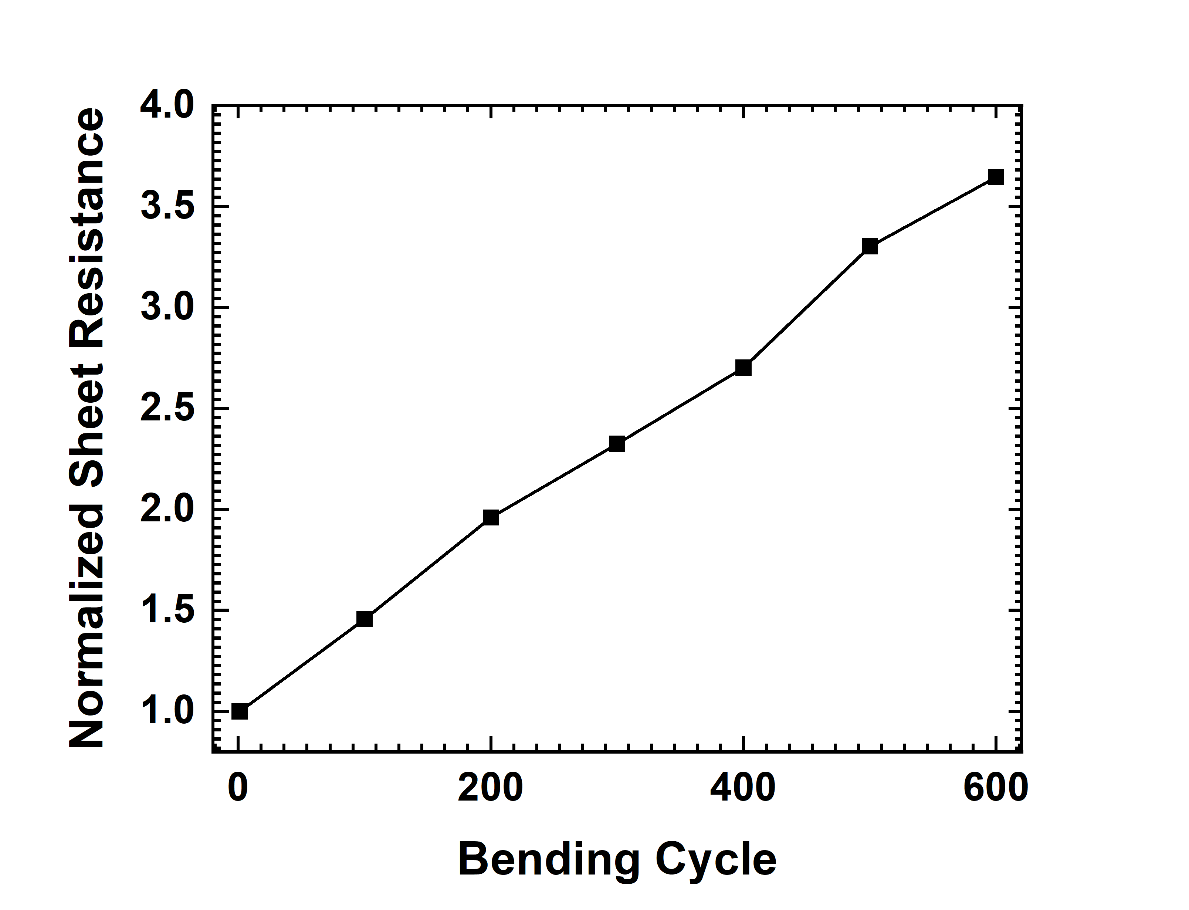


**Figure S5.** Bending cycles test results of ITO/ PEN with a bending radius of 1mm.
